# Evaluation of Immunoprotective Activities of White Button Mushroom (*Agaricus bisporus*) Water Extract Against Major Pathogenic Bacteria (*Aeromonas hydrophila* or *Vibrio fluvialis*) in Goldfish (*Carassius auratus*)

**DOI:** 10.3390/ani15152257

**Published:** 2025-08-01

**Authors:** Shujun Sun, Jing Chen, Pan Cui, Xiaoxiao Yang, Yuhan Zheng, Zijian Ma, Yong Liu, Xiang Liu

**Affiliations:** 1 Anhui Province Key Laboratory of Embryo Development and Reproductive Regulation, Fuyang Normal University, Fuyang 236041, China; 202006007@fynu.edu.cn (S.S.); 23211308@stu.fynu.edu.cn (J.C.); 23211320@stu.fynu.edu.cn (P.C.); 2024221305@fynu.edu.cn (X.Y.); 2025221324@fynu.edu.cn (Y.Z.); 2 Rural Revitalization Collaborative Technology Service Center of Anhui Province, Fuyang Normal University, Fuyang 236041, China; 201809001@fynu.edu.cn

**Keywords:** *Carassius auratus*, *Agaricus bisporus* water extract, *Aeromonas hydrophila*, *Vibrio fluvialis*, immunotherapeutic agent

## Abstract

The white button mushroom (*Agaricus bisporus*) is a widely cultivated edible and medicinal mushroom, which contains various active substances, and has application value against pathogenic bacteria in aquaculture. Firstly, *A. bisporus* water extract (AB-WE) was prepared. Through the detection kits, it was found that the polysaccharide, protein, and polyphenol components of AB-WE were 9.11%, 3.3%, and 1.5%, respectively. The 246 compounds were identified in AB-WE, and the major small-molecule components included L-Isoleucine, L-Tyrosine, L-Valine, and Linoleic acid by HPLC-Q Exactive-Orbitrap-MS. Secondly, the AB-WE was evaluated for its immunological activities through dietary administration and pathogen challenge (*Aeromonas hydrophila* and *Vibrio fluvialis*) in goldfish (*Carassius auratus*). The results showed that the levels of immune factors of acid phosphatase (ACP), alkaline phosphatase (AKP), and lysozyme (LZM) increased (*p* < 0.05) in goldfish, and the relative percentage survival of AB-WE against *A. hydrophila* and *V. fluvialis* were 80.00% (*p* < 0.05) and 81.82% (*p* < 0.05), respectively. The AB-WE reduced the bacterial content in renal tissue, enhanced the phagocytic activity of leukocytes, and exhibited antioxidant and anti-inflammatory effects by reducing the expression of antioxidant-related factors and inflammatory factors. Through histopathological and immunofluorescence techniques, it was found that AB-WE maintained the integrity of visceral tissues and reduced renal tissue apoptosis and DNA damage. Therefore, AB-WE exhibits immunoprotective activity against *A. hydrophila* and *V. fluvialis* infections in fish, and holds promise as an immunotherapeutic agent against major pathogenic bacteria in aquaculture.

## Simple Summary

*Agaricus bisporus is* a widely cultivated edible and medicinal mushroom that contains various active substances with potential applications in combating aquaculture pathogens. This study prepared an *A. bisporus* water extract (AB-WE), analyzed its main components, and evaluated its immune activities in goldfish (*Carassius auratus*) through dietary administration and pathogen challenge. The results showed that AB-WE contained polysaccharides, proteins, and polyphenols, and 38 small-molecule components were identified. Furthermore, AB-WE could activate non-specific immune activities and provided immune protection to goldfish against major aquaculture pathogens (*Aeromonas hydrophila* and *Vibrio fluvialis*). Therefore, AB-WE shows promise as an immune preparation against infections caused by major aquatic pathogens in fish.

## 1. Introduction

As a crucial pillar of global food security, the aquaculture industry has been increasingly threatened by bacterial diseases due to the promotion of intensive farming models [1,2]. Common pathogenic bacteria in aquaculture include *Aeromonas hydrophila*, *Vibrio fluvialis*, *Vibrio alginolyticus*, *Edwardsiella*, and *Pseudomonas fluorescens* [3]. In particular, *A. hydrophila* and *V. fluvialis* have caused significant economic losses in aquaculture. *A. hydrophila* is widely distributed in freshwater, sewage, and soil, and can cause various diseases such as hemorrhagic septicemia, ulcer disease, gill rot, and intestinal inflammation in fish species like crucian carp, grass carp, and perch, often leading to outbreaks that increase mortality rates in farmed fish [4]. *V. fluvialis* often attaches to residual feed at the bottom of ponds, animal feces, and dead algae. It invades the fish’s body through ingestion and surface wounds, leading to skin ulcers, redness, or spotted hemorrhages on the body surface, and intestinal inflammation [5]. These two pathogens also possess zoonotic characteristics, capable of causing sporadic diarrhea or septicemia in humans [6]. It is evident that these two aquaculture pathogens pose a threat to the sustainable development of the industry and to public health safety.

In addressing bacterial diseases in aquaculture, traditional control methods have relied heavily on antibiotics. However, the long-term misuse of antibiotics has led to issues such as bacterial resistance [7], drug residues in aquatic products [8], and the disruption of aquatic microecosystems, resulting in stringent restrictions on their use [9,10]. Vaccines, by stimulating host-specific immune responses, have emerged as a crucial direction for precise prevention and control. Some inactivated and attenuated vaccines have already achieved large-scale application in diseases such as grass carp hemorrhagic disease and shrimp white spot syndrome [11,12]. However, due to the extended time required for vaccine-induced immune activation and the susceptibility of oral vaccines to digestive degradation, their timeliness in the prevention and control of acute infections still requires optimization. Additionally, probiotics (such as *Clostridium butyricum*, *Lactobacillus*, and yeast) play a role in maintaining the balance of intestinal and aquatic microbial communities to prevent pathogenic bacterial infections in fish [13,14]. Chinese herbal medicines (such as Fuling, Banlangen, Huangqi, and Dahuang) offer advantages in the prevention and control of fish pathogens [15], including minimal toxic side effects and low drug residues. These drugs can enhance immunity in aquatic animals, improve digestion and growth, and enable effective disease prevention and treatment [16]. These benefits make them valuable for applications in aquatic animal feed, disease prevention and control, immune enhancement, and water environment improvements.

Based on the immune activity of plants, the extracts were prepared with one or more biological functions [17]. When these extracts are used as additives in aquaculture, they can promote growth and provide antioxidant and antiviral effects for fish [18]. In addition, these extracts have become one of the key research and development focuses in the industry as feed and even aquaculture water additives. Moreover, the addition of plant extracts (such as *Artemisia herbaalba*, *Isatis indigotica*, and *Astragalus membranaceus*) to the diet of *Litopenaeus vannamei* can improve protein utilization and the feed conversion ratio and promote growth and immunity [19]. Some research found that the Astragalus polysaccharides extract can enhance the ability of fish to resist bacterial infections [20]; fish fed with 100 mg/kg quercetin had significantly enhanced serum immune factors, including acid phosphatase (ACP), and lysozyme activity, as well as increased levels of C3, C4, and IgM [21]. Therefore, it is evident that plant extracts show promising potential for application in aquaculture.

White button mushroom (*Agaricus bisporus*), which belongs to the class Basidiomycetes, order Agaricales, family Agaricaceae, and genus *Agaricus*, is one of the most widely cultivated edible mushrooms globally [22]. It is rich in various bioactive substances such as polysaccharides, polyphenols, proteins, vitamins, and flavonoids, and has significant dietary and medicinal value [23]. Some studies indicate that *A. bisporus* powder can activate T cells to enhance the body’s immune response, maintain cardiovascular and cerebrovascular health by reducing blood sugar, blood pressure, and cholesterol levels, and inhibit the growth of cancer cells to exert anti-tumor effects. *A. bisporus* water extract (AB-WE) has been confirmed to be rich in β-glucans, glycoproteins, and polyphenolic compounds, showing biological activities such as antioxidant, anti-inflammatory, and immunomodulatory effects [24]. Additionally, AB-WE exerts anti-obesity effects by inhibiting pancreatic lipase-mediated lipid absorption in obese model mice [25,26]. Therefore, studies have predominantly focused on the immunomodulatory role of AB-WE in mammalian or in vitro experiments, and there is still a lack of research on its immunoprotective activity in aquaculture pathogen infection models.

**Figure 1:**
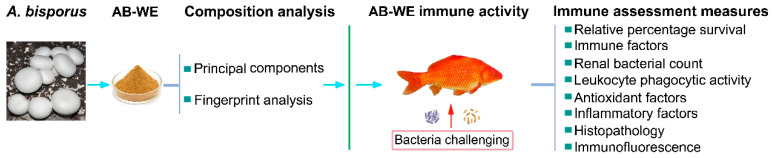
Experimental design.

This study focuses on *A. bisporus* water extract (AB-WE) as the research subject, using goldfish (*Carassius auratus*) as a carrier for evaluating drug immune effects. By feeding AB-WE to the goldfish and employing infection models of *A. hydrophila* and *V. fluvialis*, this research analyzes major indicators such as immune factors, relative percentage survival, bacterial load in visceral tissue, phagocytic activity of the leukocyte, the expression of antioxidant and inflammation factors, histopathological damage, and cell apoptosis (Figure 1). This study aims to elucidate the immune protective effects of AB-WE against major pathogens in aquaculture, providing a theoretical basis for the development of a novel green immunotherapeutic agent in aquaculture.

## 2. Materials and Methods

### 2.1. Strains and Animals

The fresh fruiting bodies of *A. bisporus* were provided by Funan Lianmei Agricultural Products Co., Ltd. (Fuyang, China). *A. hydrophila* ATCC7966, *V. fluvialis* ATCC33809, and *Staphylococcus aureus* ATCC6538 were preserved in the Biology Laboratory of Fuyang Normal University (Fuyang, China). *C. auratus* (20 ± 0.5 g) was purchased from the Fuyang Aquaculture Co., Ltd. (Fuyang, China). All animal experiments were conducted in accordance with the Guide for the Care and Use of Laboratory Animals, and approval was received from the Institutional Animal Care and Use Committee of Fuyang Normal University, China (No. 2024-04-015).

### 2.2. Preparation of A. bisporus Water Extract

*A. bisporus* water extract (AB-WE) was prepared according to the methods of He et al. [27]. Briefly, 1 kg of *A. bisporus* was freeze-dried, ground, and subsequently passed through a 60-mesh sieve. Deionized water was then added at a solid-to-liquid ratio (mass/volume) of 1:40, and the mixture was extracted for 3 h in a water bath at 80 °C. The resulting extract was centrifuged at 4 °C and 4500 r/min for 10 min, and the supernatant was collected. This extraction process was repeated twice, and the supernatants from both extractions were combined. The combined supernatant was dried using rotary evaporation followed by freeze-drying, yielding AB-WE powder with an extraction rate of approximately 45%.

### 2.3. AB-WE Component Analysis

The AB-WE polysaccharide, protein, and polyphenol components were measured according to the kit instructions (Sangon Biotechnology Co., Ltd., Shanghai, China).

### 2.4. Fingerprint Analysis of Small-Molecule Compounds in AB-WE

Fingerprint analysis was conducted according to the previous experimental method [28]. Briefly, AB-WE was preliminarily analyzed using the HPLC-Q Exactive-Orbitrap-MS (Thermo Fisher Scientific, Waltham, MA, USA). Chromatographic separation was performed using the Ultimate 3000 HPLC system (2.1 mm × 150 mm × 1.8 μm) column (Welch, IL, USA). The mobile phase comprised (A) 0.1% methanol in pure water and (B) methanol with gradient elution (98% A for 1 min, decreased to 80% A over 4 min, decreased to 50% A over 5 min, decreased to 20% A over 5 min, decreased to 5% A over 5 min, and maintained at this phase for 7 min, then increased to 98% A over 2 min, and maintained at this phase for 2 min). The samples were maintained at room temperature and the injection volume was 5 μL. The Q Exactive mass spectrometer equipped with a heated electrospray ionization interface was operated under both electrospray ionization (ESI)-negative and ESI-positive modes. The instrument was calibrated with the calibration solutions provided by the manufacturer. Data were acquired using Compound Discoverer 3.3 software (Thermo Fisher Scientific, Waltham, MA, USA), and the preliminary sorting data were searched and compared in the database (mzCloud). The other parameters were set as follows: source parameters, spray voltage of 3.2 kV (+)/3.2 kV (−); capillary temperature, 300 °C; auxiliary gas heater temperature, 350 °C; sheath gas pressure, 40 arb; and auxiliary gas pressure, 15 arb. The data were statistically analyzed using SPSS 19.0 software.

### 2.5. Median Lethal Dose (LD_50_) of A. hydrophila or V. fluvialis in C. auratus

The *C. auratus* were divided into two major groups for challenging with *A. hydrophila* or *V. fluvialis*, respectively. Each major group was further divided into four subgroups, with 10 fish in each subgroup. Each group of fish was fed a basal diet without AB-WE. The basal diet for goldfish was 2 mm diameter fish pellet feed (Fuyang Aquaculture Co., Ltd., Fuyang, China). The goldfish were fed twice daily, with 0.1 g of feed per fish each time. The fish were fed once a day for 10 continuous days. After the last feeding, the fish were intraperitoneally challenged with a dose of 40 μL of *A. hydrophila* (4 × 10^8^, 8 × 10^8^, 10 × 10^8^, 12 × 10^8^ CFU) or *V. fluvialis* (6 × 10^8^, 8 × 10^8^, 1 × 10^9^, 2 × 10^9^ CFU), respectively. Four days later, the LD_50_ of *A. hydrophila* or *V. fluvialis* was determined by calculating the bacterial dose at which half of the fish died. The results showed that the LD_50_ of *A. hydrophila* or *V. fluvialis* were 8 × 10^8^ CFU and 1 × 10^9^ CFU, respectively.

### 2.6. Relative Percentage Survival of AB-WE in Fish

The goldfish (20 ± 0.5 g) were divided into two major groups, which were challenged with *A. hydrophila* and *V. fluvialis*, respectively. Each major group was further divided into two subgroups, with 15 fish per group. Within the two subgroups, one group was fed sterile water without AB-WE as the nature control group (NC), and the other group was fed with AB-WE. Then, AB-WE was dissolved in water to prepare a 0.125 g/mL solution, which was sterilized using a 0.22 μm pore size filter. The goldfish in the AB-WE group and the control group were fed the basal diet, which was a 2 mm diameter fish pellet feed (Fuyang Aquaculture Co., Ltd., Fuyang, China), with 0.1 g of feed per fish each time. Subsequently, the goldfish in the AB-WE group were administered 40 μL of AB-WE solution (5 mg per fish) (at a ratio of 250 μg/g of AB-WE to fish mass) using an animal feeding needle, while the control group received 40 μL of sterile water per fish. The fish were fed once a day for 10 continuous days. After the last feeding instance with AB-WE, the fish were challenged with *A. hydrophila* (8 × 10^8^ CFU) or *V. fluvialis* (1 × 10^9^ CFU) according to preliminary experiments, respectively. Following the challenge, mortality was observed for 14 days. The relative percentage survival (RPS) was calculated using the formula RPS (%) = (1 − [experimental group mortality %/control group mortality %]) × 100 [29]. The data were statistically analyzed using SPSS 19.0 software.

### 2.7. Immune Factor Analysis

On the 10th day of feeding fish with AB-WE, blood samples were collected from the tail vein of the fish under anesthesia. Serum samples were obtained with 3000 r/min centrifugation at 4 °C for 15 min. The levels of immune factors of acid phosphatase (ACP), alkaline phosphatase (AKP), and lysozyme (LZM) were evaluated according to the instructions provided by the detection kit (Sangon Biotechnology Co., Ltd., Shanghai, China).

### 2.8. Renal Bacterial Count

Goldfish were continuously fed with AB-WE for 10 days, while a control group was fed a basal diet without AB-WE as natural control (NC). Then the goldfish were challenged with *A. hydrophila* or *V. fluvialis*. Additionally, another group of goldfish was fed a basal diet without AB-WE and was not exposed to any infection bacteria, serving as a negative control. Two days later, kidney tissues were collected and homogenized under aseptic conditions with 400 μL of phosphate-buffered saline (PBS). To count bacterial colonies, the kidney homogenate was evenly spread on the surface of the LB medium and incubated overnight at 37 °C in a constant-temperature incubator. Then, the bacterial colonies on the LB medium were counted to evaluate the inhibitory effect of AB-WE on *A. hydrophila* and *V. fluvialis* in fish [30].

### 2.9. Leukocyte Phagocytic Activity

The fish were fed with AB-WE and then challenged with *A. hydrophila* or *V. fluvialis*. Two days later, the plasma was collected from the tail vein under anesthesia. Meanwhile, *S. aureus* was inactivated with 1% formaldehyde at 80 °C for 90 min. Then, 0.2 mL of plasma was mixed with 0.2 mL *S. aureus* (7 × 10^8^ CFU/mL), and the mixture was incubated in a water bath at 25 °C for 60 min. After incubation, the mixture was dropped onto a glass slide and evenly spread to create a blood smear. The blood smear was stained using a rapid Giemsa staining kit (Sangon Biotech Co., Ltd., Shanghai, China), and the count of phagocytic cells was conducted with a microscope (Leica, Wetzlar, Germany). The phagocytic rate (*PR*%) was calculated using the following formula: *PR*% = (number of cells participating in phagocytosis among 100 phagocytes/100) × 100%. Additionally, the phagocytic index (*PI*) was calculated as follows: *PI* = (number of bacteria in phagocytes/number of cells participating in phagocytosis) [3]. Data were statistically analyzed using SPSS 19.0 software.

### 2.10. Antioxidant Factor Analysis

The fish were fed with AB-WE and then challenged with *A. hydrophila* or *V. fluvialis*. Two days later, the plasma was collected from the tail vein under anesthesia. The levels of antioxidant factors of superoxide dismutase (SOD), catalase (CAT), malondialdehyde (MDA), and glutathione peroxidase (GSH-Px) were evaluated following the instructions provided by the detection kits (Sangon Biotechnology Co., Ltd., Shanghai, China).

### 2.11. mRNA Expression of Inflammatory Factors in Visceral Tissues

The expression of inflammatory factor mRNA was assessed using the real-time quantitative PCR method (qRT-PCR). Briefly, after goldfish were fed with AB-WE and challenged with pathogenic bacteria, and the kidneys and spleens collected on the second day under anesthesia, the tissues were thoroughly ground with liquid nitrogen, and total RNA was extracted according to the RNA extraction kit instructions (Takara, Beijing, China). The RNA was then reverse-transcribed into cDNA following the instructions of the reverse transcription kit (Takara, Beijing, China). qRT-PCR was performed using the SYBR^®^ Green Premix kit (Takara, Beijing, China) and synthesized primers (Supplementary Table S1). Then, the ΔCt (cycle threshold change) was calculated by comparing the Ct value of the target gene with that of the internal reference gene (*gapdh*). Subsequently, the difference in ΔCt values between the experimental and control groups was used to derive the ΔΔCt. Finally, the 2^−(ΔΔCt)^ formula was applied to analyze mRNA expression [29]. Data were statistically analyzed using SPSS 19.0 software.

### 2.12. Histopathological Analysis of Visceral Tissues

Histopathological analysis was conducted according to the previous experimental method [30]. Briefly, the fish were fed with AB-WE and then challenged with *A. hydrophila* or *V. fluvialis*. Two days later, kidney, spleen, and intestinal tissues were collected under anesthesia, and immersed in Davidson’s fixative and 10% formaldehyde solution for 24 h for fixation, respectively. Following fixation, the tissues were dehydrated through a graded ethanol series (70%, 80%, 90%, 95%, and 100% ethanol), then cleared with xylene twice, each time for 30 min. Then, the cleared tissues were embedded in paraffin at 60 °C and sectioned into 4 μm thick slices using a microtome (Leica, Wetzlar, Germany). The sections were placed on glass slides and dried on a slide warmer at 60 °C for 3 h. Subsequently, the sections were deparaffinized with xylene, dehydrated through a graded ethanol series, and cleared with xylene, followed by staining with hematoxylin and eosin (H&E). After being cleared with xylene, the sections were observed and photographed under a microscope (Leica, Wetzlar, Germany).

### 2.13. Renal Immunofluorescence Analysis

The renal immunofluorescence analysis was conducted following the previous experimental protocol [3]. Briefly, the prepared renal tissue sections were dewaxed in xylene and then hydrated in a decreasing gradient of ethanol concentrations. After antigen retrieval solution treatment, the tissue periphery was circled with an immunohistochemical pen, and 50 μL of 5% bovine serum albumin (BSA) blocking solution was added within the circle to block the samples at 25 °C for 2 h. After the sections were washed with PBST, monoclonal antibodies against p53 or γH2A.X (dilution ratio 1:500) were added to the tissue and incubated overnight at 4 °C. After washing, the secondary antibody solution (dilution ratio 1:1000) was added and incubated at 37 °C for 1 h. After washing, the sections were stained with the nuclei with 4′,6-diamidino-2-phenylindole (DAPI) at room temperature in the dark for 10 min, and photographs were taken under a fluorescence microscope (Leica, Wetzlar, Germany).

### 2.14. Statistical Analysis

All the experimental data were expressed as mean ± SD, and all experiments were repeated at least three times. The significant difference from the respective control in all experiments was assessed by one-way analysis of variance (ANOVA) using Statistical Package for the Social Sciences 19.0 (SPSS19.0) software. Values of *p* < 0.05 were considered statistically significant [3].

## 3. Results

### 3.1. Component Analysis of AB-WE

Based on the test with the reagent kit, the AB-WE’s polysaccharide, protein, and polyphenol components were 9.11%, 3.3%, and 1.5%, respectively. Polysaccharides showed a higher proportion compared to the other substances (Table 1).

### 3.2. Fingerprint Analysis of AB-WE Using HPLC-Q Exactive-Orbitrap-MS

**Figure 2:**
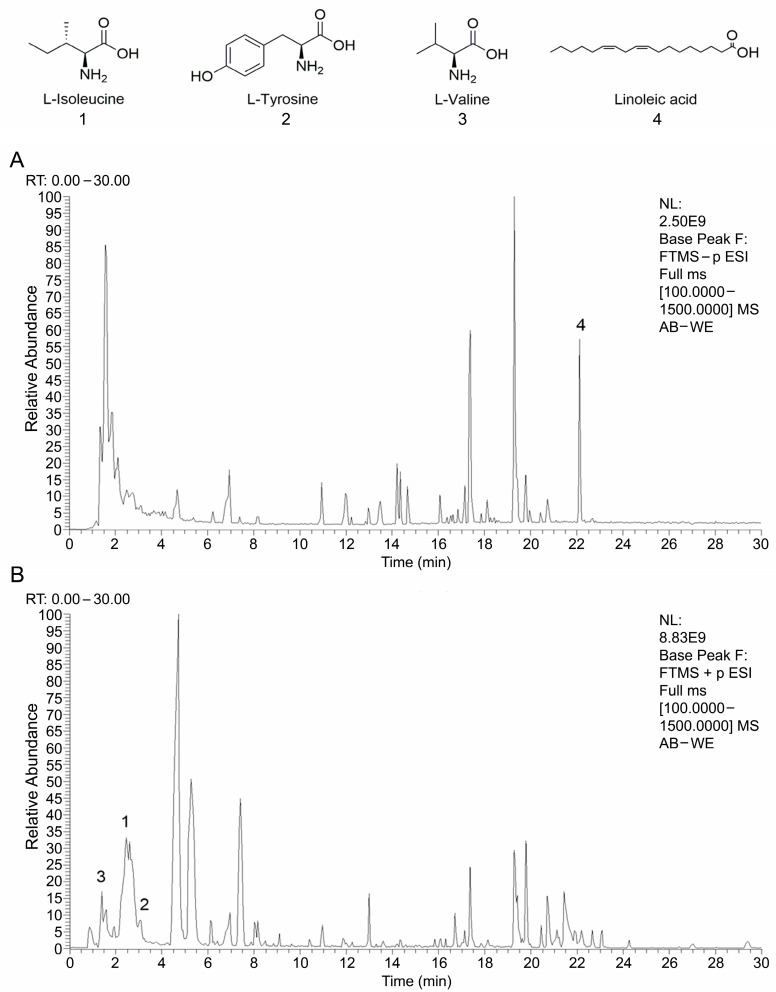
The fingerprint of AB–WE using an HPLC–Q Exactive–Orbitrap–MS instrument. (**A**,**B**) The map from ESI–negative Ion Mode and ESI–positive Ion Mode. The peaks labeled 1, 2, 3, and 4 correspond to L–Isoleucine, L–Tyrosine, L–Valine, and Linoleic acid, respectively. These four compounds were identified with a matching degree > 85% in mzCloud and ranked as the top four based on peak area, from largest to smallest.

AB-WE was analyzed using HPLC-Q Exactive-Orbitrap-MS. The data acquired from the HPLC-Q Exactive-Orbitrap-MS instrument were initially processed using Compound Discoverer 3.3 (CD 3.3, Thermo Fisher) and subsequently compared against the mzCloud database. In total, 246 compounds were identified in mzCloud, with 38 compounds achieving a comprehensive score of mzCloud best match exceeding 85% (Supplementary Table S2). The fingerprint of AB-WE is shown in Figure 2. The major small-molecule components identified in AB-WE, L-Isoleucine, L-Tyrosine, L-Valine, and Linoleic acid, are clearly annotated in the fingerprint chromatogram (Figure 2), and they were the top four compounds based on peak area among the compounds with a matching degree > 85% in mzCloud. In the chromatogram, several peaks exhibiting large peak areas but low matching scores were observed. These unidentified compounds are predominantly high-molecular-weight substances, primarily comprising carbohydrates and nitrogen-containing organic compounds such as peptides (Figure 2). This suggests that the sample still contains a notable quantity of polysaccharides or oligopeptides that were not identified by HPLC-Q Exactive-Orbitrap-MS.

### 3.3. Immunoprotective Abilities of AB-WE Against Pathogenic Bacteria

To determine the bacterial challenge dose, the LD_50_ of *A. hydrophila* and *V. fluvialis* was performed. The results showed that the LD_50_ of *A. hydrophila* and *V. fluvialis* were 8 × 10^8^ CFU and 1 × 10^9^ CFU (Supplementary Table S3), respectively.

**Figure 3:**
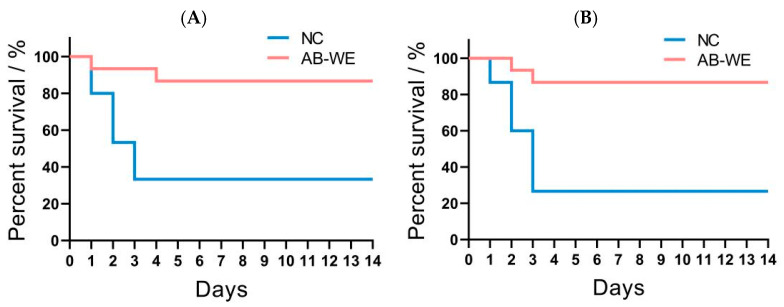
The goldfish survival rate curve. (**A**,**B**) Challenge with *A. hydrophila* and *V. fluvialis*, respectively. (NC) Blank IgY as nature control.

To investigate relative percentage survival (RPS)of AB-WE against pathogenic bacterial infections, the goldfish were fed with AB-WE, and then challenged with *A. hydrophila* or *V. fluvialis*. The results showed that the fish exhibited symptoms such as slow swimming, epidermal hemorrhage, and abdominal swelling after the challenge. The control group experienced significant mortality, with the death rate stabilizing after 4 days (Figure 3). Additionally, the relative percentage survival of goldfish treated with AB-WE against *A. hydrophila* and *V. fluvialis* was 80.00% (*p* < 0.05) and 81.82% (*p* < 0.05), respectively (Table 2). It is evident that AB-WE can provide immune protection for goldfish against infections caused by *A. hydrophila* and *V. fluvialis*.

### 3.4. Immune Factors Analysis

**Figure 4:**
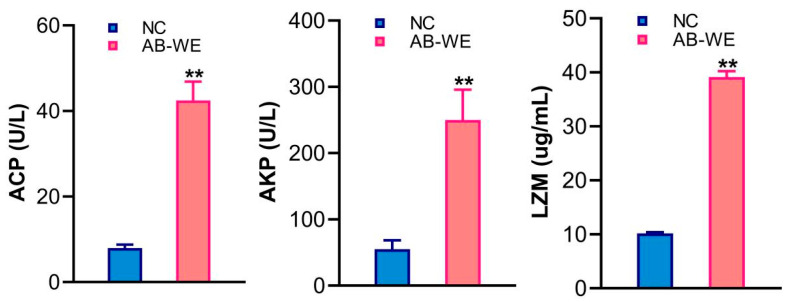
The expression levels of ACP, AKP, and LZM in the serum of goldfish. ** *p* < 0.01 (compared with control).

To analyze the immune activation effect of AB-WE on goldfish, the immune factors in the serum were detected after fish had been fed AB-WE. The results showed that compared with the control group, the levels of ACP, AKP, and LZM in the serum of the AB-WE group were significantly increased (*p* < 0.01) (Figure 4), indicating that AB-WE has an immune activation effect.

### 3.5. Bacterial Count in the Kidney of Goldfish

**Figure 5:**
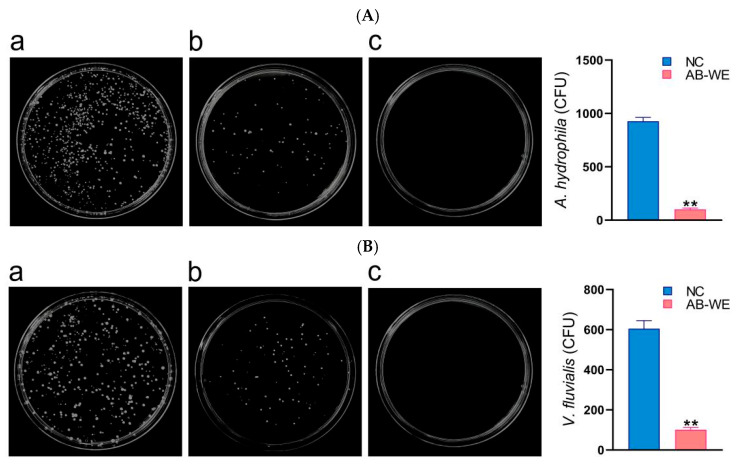
Bacterial content in the goldfish kidney. (**A**,**B**) Challenge with *A. hydrophila* or *V. fluvialis*, respectively; (**a**) no treatment with AB-WE, as the natural control (NC); (**b**) fed with AB-WE; and (**c**) kidney samples without bacterial infection as the negative control. ** *p <* 0.01 (compared with the NC group).

To assess the bacterial quantity within the goldfish, kidney tissues were collected and coated in LB medium for the bacterial colony count. The results showed that compared with the control group, the bacterial quantity in the kidneys of goldfish immunized with AB-WE decreased (*p* < 0.05) (Figure 5). The result indicates that AB-WE can eliminate bacteria in the kidneys, demonstrating that AB-WE possesses immune activity.

### 3.6. Phagocytic Activity of Leukocytes in Goldfish

To evaluate the phagocytic activity, the goldfish were fed with AB-WE and challenged to pathogenic bacteria, and the plasma of the goldfish was collected for a phagocytosis assay of leukocytes. The results showed that both the phagocytic rate (*PR*%) and the phagocytic index (*PI*) of leukocytes increased (*p* < 0.05) in the AB-WE group compared with the control group (Table 3), indicating that AB-WE can activate the phagocytic activity of leukocytes in goldfish.

### 3.7. Antioxidant-Related Factors in Goldfish

**Figure 6:**
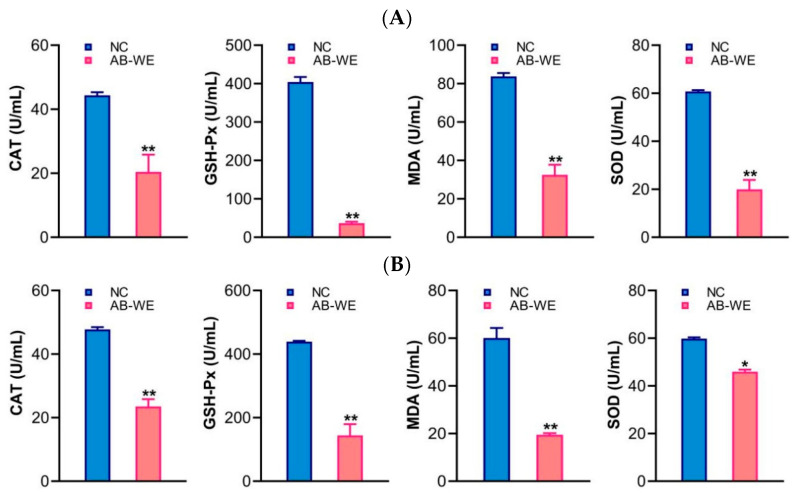
Detection of antioxidant factor content (CAT, SOD, MDA, and GSH-Px) in serum. (**A**,**B**) Challenge with *A. hydrophila* or *V. fluvialis*, respectively. * *p* < 0.05 and ** *p* < 0.01 (compared with control).

After feeding goldfish with AB-WE and challenging them with pathogenic bacteria, the antioxidant factors were evaluated in sera. The results showed that, compared with the control group, after challenging with *A. hydrophila* or *V. fluvialis*, the antioxidant-related factors (CAT, GSH-Px, MDA, and SOD) were significantly decreased (*p* < 0.05) in the serum of goldfish fed with AB-WE (Figure 6). The results indicate that AB-WE can reduce the oxidative stress response caused by bacterial infection, and that it possesses antioxidant activity.

### 3.8. mRNA Expression of Inflammation-Related Genes in Goldfish Visceral Tissues

**Figure 7:**
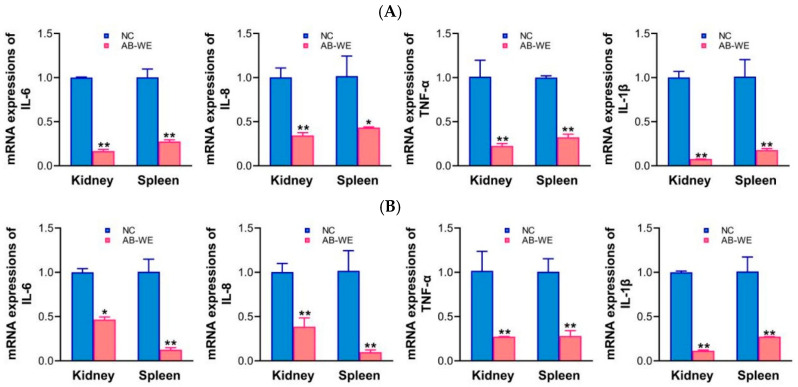
Inflammatory factor mRNA expression by qRT-PCR analysis. (**A**,**B**) Challenge with *A. hydrophila* or *V. fluvialis*, respectively. * *p* < 0.05 and ** *p* < 0.01 (compared with control).

After the goldfish were fed with AB-WE and challenged with *A. hydrophila* or *V. fluvialis*, the kidneys and spleens were collected to assess the mRNA expression of inflammation-related genes. The results showed that the mRNA expression levels of inflammatory factors (IL-6, IL-8, TNF-α, and IL-1β) were significantly reduced (*p* < 0.05) in the kidneys and spleens of the AB-WE group compared with the control group (Figure 7). The results indicate that AB-WE can alleviate the inflammatory response caused by bacterial infection in goldfish, demonstrating that AB-WE shows anti-inflammatory activity.

### 3.9. Histopathological Morphological Observation of Goldfish Visceral Tissues

**Figure 8:**
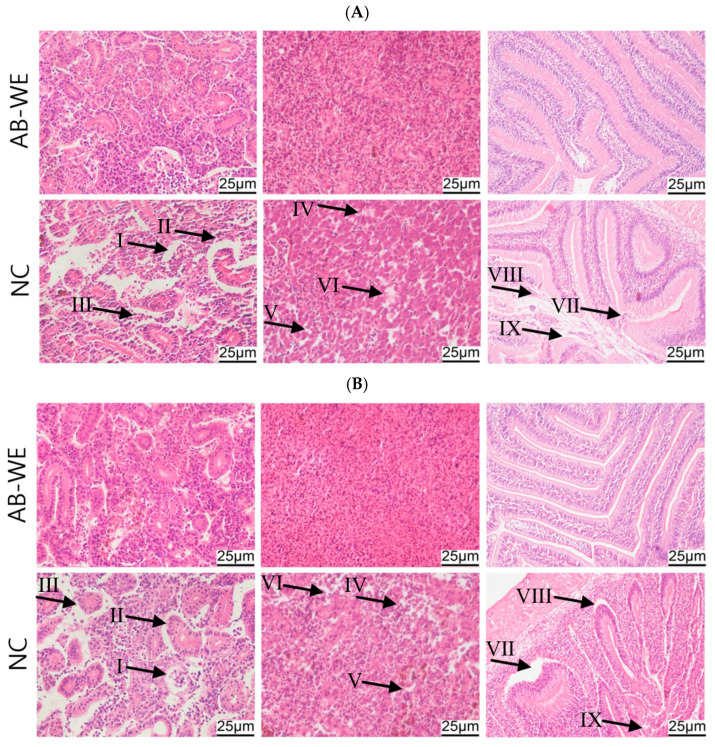
Histopathological sections of the kidney, spleen, and intestinal tissues of goldfish. (**A**,**B**) Challenge with *A. hydrophila* or *V. fluvialis*, respectively. (I) Glomerular atrophy; (II) Loose renal tubular; (III) Renal cell apoptosis; (IV) Low density of splenic cells; (V) Splenic cell apoptosis; (VI) Incomplete structure of splenic tissue; (VII) Intestinal villus necrosis; (VIII) Intestinal mucosal necrosis; (IX) Intestinal cell apoptosis.

To evaluate the structural damage in the visceral tissues, the fish were fed AB-WE and challenged with *A. hydrophila* and *V. fluvialis*. Then, kidney, spleen, and intestinal tissues were collected to prepare histopathological sections. The results showed that, in the control group, the goldfish kidney tissues were loose and incomplete, with atrophy and apoptosis of the glomeruli and renal tubules. Additionally, the spleen tissues were also incomplete, showing reduced cell density and cellular apoptosis. Further, the intestinal mucosal villi were atrophied, with incomplete structures and cellular apoptosis. In contrast, in the AB-WE group, the kidney, spleen, and intestinal tissues of goldfish maintained intact and clear structures with no significant pathological damage observed (Figure 8). These results indicate that AB-WE can alleviate visceral tissue damage caused by pathogen infection in goldfish.

### 3.10. Renal Tissue Cell Apoptosis and DNA Damage in Goldfish

**Figure 9:**
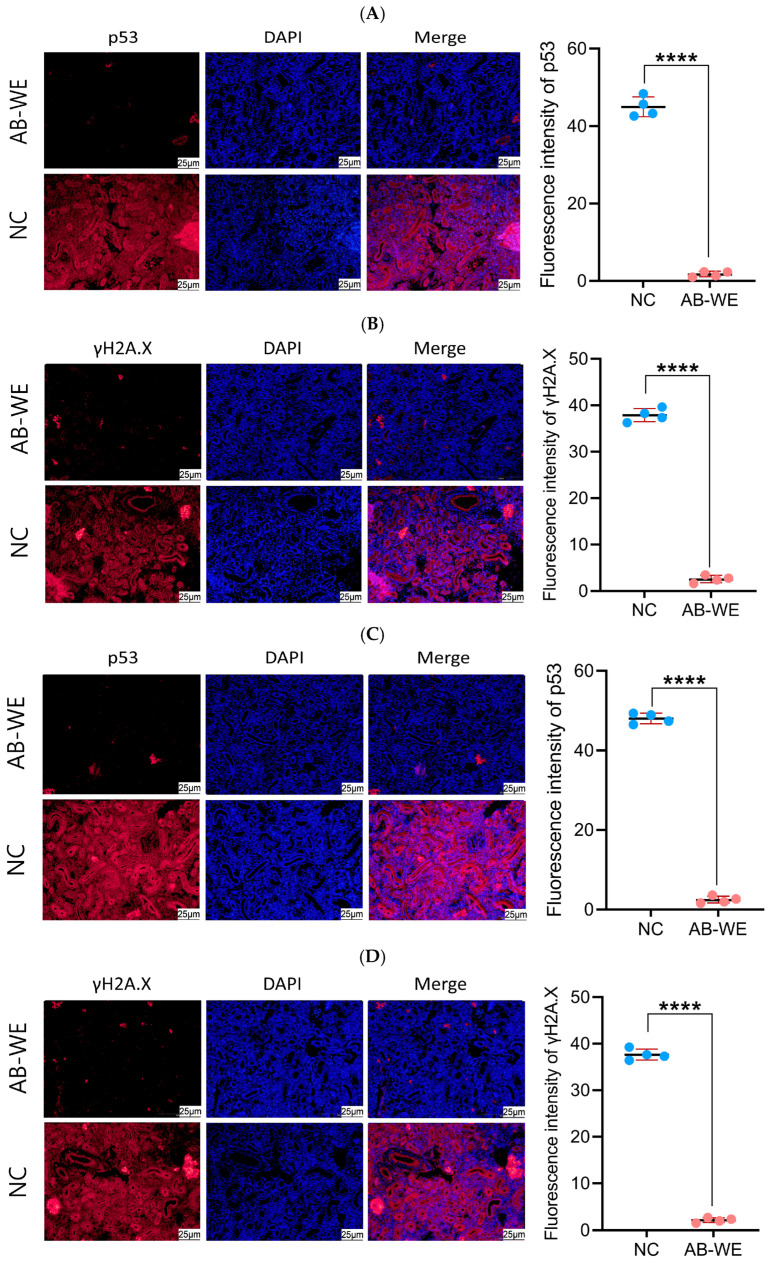
The expressing of p53 and γH2A.X in the goldfish kidney according to immunofluorescence analysis. (**A**,**B**) Challenge with *A. hydrophila*. (**C**,**D**) Challenge with *V. fluvialis*. (**A**,**C**) p53 immunofluorescence. (**B**,**D**) γH2A.X immunofluorescence. **** *p* < 0.0001 (compared with control).

To evaluate apoptosis and DNA damage in the kidney cells of goldfish, immunofluorescence analysis of the apoptotic factor (p53) and DNA damage factor (γH2A.X) was conducted using kidney tissue histopathological sections. The red fluorescence indicates p53 and γH2A.X proteins, while the blue fluorescence indicates the cell nuclei. The results showed that, compared to the control group, the expression levels of p53 and γH2A.X were significantly reduced (*p* < 0.05) in the kidney tissues of the AB-WE group (Figure 9). The results indicate that AB-WE can reduce apoptosis and DNA damage caused by pathogenic bacterial infection in the goldfish kidney cells.

## 4. Discussion

As a widely consumed food ingredient, the edible portion (fruiting body) of *A. bisporus* is regarded as a traditional food globally, and it has been safely consumed as a daily dietary component for a long time. According to the FDA’s regulatory framework, the fruiting bodies of conventionally consumed natural ingredients, when used as whole foods, are presumed to have a Generally Recognized as Safe (GRAS) status by default, and do not require additional GRAS notifications or specific certifications. In this study, the *A. bisporus* water extract was derived from the fruiting bodies of conventional edible strains, and its preparation method aligns with traditional food processing practices, meeting the relevant GRAS safety requirements of. In this study, water was chosen as the extraction method for the active components of *A. bisporus* because water is an inexpensive solvent, which reduces the costs for large-scale production. Water extract is highly safe without harmful substances, and the operation process is relatively simple. It also offers better extraction efficiency for many hydrophilic components such as polysaccharides, proteins, and polyphenols [31,32]. Khalil et al. performed a hot water extraction protocol at 90 °C for 5 h with a liquid-to-solid ratio of 118.59 mL/g, which yielded a polysaccharide content of 20.9% [32]. In this study, AB-WE was prepared via hot water extraction at 80 °C for 3 h with a liquid-to-solid ratio of 40 mL/g, yielding a polysaccharides content of 9.11%. The polysaccharide content of AB-WE was therefore lower in this research compared to the one in Khalil’s study. This might be due to the previous study’s extraction conditions, which involved higher temperature, longer duration, and more reagent multiples. However, the advantage of this study is that the polysaccharides may exhibit less degradation and higher activity. In addition, most experiments on AB-WE only conducted polysaccharide content detection [33]. In this research, the proportions of protein and polyphenol in AB-WE were 3.3% and 1.5%, respectively, and these substances may be related to immune activity. Additionally, in this study, the fingerprint map of AB-WE was analyzed using HPLC-Q Exactive-Orbitrap-MS, and the four compounds with the highest content proportions in AB-WE are L-isoleucine, L-tyrosine, L-valine, and linoleic acid. Isoleucine and valine are branched-chain essential amino acids that cannot be synthesized by the human body. Animal studies have shown that a deficiency in the intake of branched-chain amino acids such as leucine, isoleucine, and valine can lead to damage to the immune system [34]. It has been reported that the dietary restriction of isoleucine and valine resulted in an approximately 80% reduction in lymphocyte-mediated tumor cell lysis [35]. Meanwhile, tyrosine, a breakdown product of phenylalanine, is a direct precursor in the synthesis of adrenaline, noradrenaline, and thyroid hormones, as well as dopamine and melanin [36]. Norepinephrine is a key messenger released by the sympathetic nervous system, and it acts directly on the immune system [37]. Studies have found that a deficiency of phenylalanine and tyrosine in the diet of chickens weakens the immune response, and supplementing these two substances in the diet can reverse this condition [38]. Linoleic acid can modulate inflammatory responses through eicosanoids, enhancing immunity at low and medium doses [39]. Therefore, AB-WE was obtained using the water extraction method, laying the foundation for immune function evaluations.

There have been few research reports on the direct application of *A. bisporus* water extract in the prevention and control of diseases in aquaculture. However, the waste material (mushroom bran/mushroom residue) of *A. bisporus* can serve as a potential resource in aquaculture, including in the preparation of raw materials for aquatic feed, the improvement of water quality and substrate in aquaculture, and the cultivation of aquatic bait organisms, and it holds significant potential in ecological recycling aquaculture models. Research has shown that feeding bees with AB-WE can enhance their resistance to *Nosema ceranae* infection [33]. When T-helper 1 (Th1) cells were cultured with AB-WE, an increase in the expression of immune-related factors (interferon-gamma (IFN-γ) and interleukin (IL)-4) was observed, indicating that the AB-WE activated the immune activity of T cells [40]. Additionally, Yang et al. fed channel catfish with polysaccharides from *A. bisporus* and observed an enhancement in the expression of immune-related factors (IL-1β, Hsp70, and IgM); when the feeding content of *A. bisporus* polysaccharides exceeded 125 mg/kg, the protection rate against *Yersinia ruckeri* bacterial infection in fish exceeded 60% [41]. Interestingly, the addition of 1% *A. bisporus* powder to zebrafish feed increased the expression of immune-related factors (lysozyme) in the skin and intestine, indicating that *A. bisporus* powder has an immunostimulatory effect [42]. Moreover, mixing *A. bisporus* powder with lactic acid bacteria can further activate the immune activity of *A. bisporus* powder in fish [43]. In this study, after the goldfish was fed with AB-WE and challenged with pathogenic bacteria, it was found that the relative percentage survival of AB-WE against *A. hydrophila* and *V. fluvialis* were 80.00% (*p* < 0.05) and 81.82% (*p* < 0.05), respectively. Furthermore, the expression levels of immune factors increased in serum, the bacterial count in the kidneys decreased, and the bacterial phagocytic activity of the white blood cells was enhanced. The results indicate that AB-WE can activate the immune activity of goldfish to resist major pathogens in aquaculture.

The antioxidant effect refers to a reduction in oxidative damage by neutralizing free radicals, thereby helping delay aging and prevent animal diseases [44]. The experimental detection of antioxidant indicators includes the scavenging of free radical content and the expression of antioxidant-related factors (CAT, SOD, MDA, and GSH-Px). Khalil et al. discovered that the water extraction method is a simple and effective technique for extracting polysaccharide components from *A. bisporus*, and that AB-WE exhibits DPPH, ABTS, and hydroxyl radical scavenging activities, indicating its antioxidant properties [32]. Udeh et al. found that the cold-water extract of *A. bisporus* exhibited higher scavenging capacities against DPPH and hydrogen peroxide than hot-water extract or ethanol extract [45]. This study found that after feeding goldfish with AB-WE and challenging them with pathogenic bacteria, antioxidant-related factors (CAT, SOD, MDA, and GSH-Px) decreased in serum, indicating that AB-WE reduced the oxidative stress response caused by bacterial infection in goldfish, suggesting that AB-WE possesses antioxidant activity.

Anti-inflammatory activity testing reflects the ability of an animal to resist pathogenic bacterial infections, and it can be evaluated by detecting the expression of inflammation-related factors to assess the animal’s anti-inflammatory capacity [46]. Smiderle et al. prepared an inflammatory cell model using bacterial LPS in THP-1 cells, and then added AB-WE to the inflammatory cells. They found that the expression of inflammatory factors (IL-1β, TNF-α, COX-2) in THP-1 cells was reduced, indicating that AB-WE possesses anti-inflammatory activity [47]. Further, Yang et al. fed a catfish virus model with *A. bisporus* polysaccharides and found that the polysaccharides could reduce the expression of inflammation-related factors (IL-6, IFN-α3, IFN-γ1, IL-26, Casp3, Casp8, IL-10, NLRC3), indicating that *A. bisporus* polysaccharides can modulate inflammation to enhance the antiviral capacity of catfish [48]. Additionally, Gallego et al. utilized AB-WE to treat non-alcoholic steatohepatitis (NASH) in rats, and found that feeding with AB-WE could increase the expression of anti-inflammatory factors (PPARα, TLR4), thereby achieving anti-inflammatory effects [49]. In this study, after the administration of AB-WE to goldfish and following pathogen infection, the expression of inflammation-related factors (IL-6, IL-8, TNF-α, and IL-1β) displayed a significant reduction (*p* < 0.05) in visceral tissues. This indicates that AB-WE can mitigate the inflammatory response induced by pathogen infection in goldfish, demonstrating its anti-inflammatory properties.

The intact structure and function of cells are crucial for maintaining animals’ resistance to pathogenic infections [50]. Therefore, histopathology can be utilized to assess the integrity of cellular structures, thereby evaluating the immunoprotective activity of drugs on the animal body [51]. Dimopoulos et al. constructed a mouse model for Alzheimer’s disease and fed the mice a diet containing 10% *A. bisporus* powder. Subsequently, they prepared pathological sections of the mouse brain tissue, and observed a reduction in plaque deposition on the hippocampus [52]. Kanaya et al. utilized liver histopathological sections and discovered that feeding mice with *A. bisporus* powder could reduce liver fat accumulation, and *A. bisporus* powder provided a protective effect against hepatic steatosis in mice [53]. In this study, goldfish were fed AB-WE and then challenged with pathogenic bacteria. A histopathological examination of fish tissues revealed intact pathological structures in the kidney, spleen, and intestinal tissues. Additionally, immunofluorescence analysis of the pathological section showed a reduction in the expression of the apoptotic factor (p53) and the DNA damage factor (γH2A.X) in kidney tissues. Thus, AB-WE can protect the structural integrity of visceral tissues caused by pathogenic bacterial infections in fish.

## 5. Conclusions

This study prepared AB-WE and evaluated its immunoprotective effects by feeding it to goldfish, and then challenging the fish with pathogenic bacteria. The results showed that AB-WE contained polysaccharides, proteins, and polyphenols, and 246 compounds were identified. Meanwhile, AB-WE demonstrated immunoprotective effects against pathogenic infections, activated the levels of immune factors, cleared pathogens from the kidney, enhanced the phagocytic activity of leukocytes, and improved the antioxidant and anti-inflammatory capacities in goldfish. Further, AB-WE could maintain the integrity of visceral tissues and reduce apoptosis and DNA damage to the kidney. Therefore, AB-WE can be used as an immune preparation against major pathogenic bacteria in fish.

## Figures and Tables

**Table 1:** The component analysis of AB-WE.

No.	Chemical Composition	Content Ratio (%)
1	Polysaccharides	9.11 ± 0.54
2	Proteins	3.3 ± 0.37
3	Polyphenols	1.5 ± 0.09

**Table 2:** The relative percentage survival of goldfish treated with AB-WE.

Bacteria	Group	No.	Survival, No.	Death, No.	ADR, %	RPS, %
*A. hydrophila*	NC	15	5	10	66.67	--
	AB-WE	15	13	2	13.33	80.00 **
*V. fluvialis*	NC	15	4	11	73.33	--
	AB-WE	15	13	2	13.33	81.82 **

Notes: ADR, accumulating death rate. RPS, relative percentage survival. RPS (%) = 1 − (% AB-WE mortality/% non-AB-WE mortality) × 100. ** *p* < 0.01 (compared with control). NC represents the fish feed basal diet without AB-WE as the nature control group.

**Table 3:** Phagocytic activity of goldfish leukocytes.

Challenge Bacteria	Group	Phagocytic Rate (*PR*%)	Phagocytic Index (*PI*)
*A. hydrophila*	NC	25.36 ± 4.95	68.34 ± 8.67
	AB-WE	70.36 ± 8.34 **	150.36 ± 10.31 **
*V. fluvialis*	NC	38.45 ± 14.37	80.14 ± 13.17
	AB-WE	73.28 ± 5.39 **	140.78 ± 13.49 **

** *p* < 0.01 (compared with control).

## Data Availability

The data presented in this study are available on request from the corresponding author.

## Supplementary Materials

The following supporting information can be downloaded at https://www.mdpi.com/article/10.3390/ani15152257/s1, Table S1: Primers used for the qRT-PCR; Table S2: Structural characterization of the identified compounds; Table S3: The LD_50_ determination of *A. hydrophila* and *V. fluvialis* in *C. auratus*.
