# Supplementary material for: Evaluation of Immunoprotective Activities of White Button Mushroom (Agaricus bisporus) Water Extract Against Major Pathogenic Bacteria (Aeromonas hydrophila or Vibrio fluvialis) in Goldfish (Carassius auratus)
Source: Animals (Basel). 2025 Aug 1;15(15):2257. doi: 10.3390/ani15152257 (PMC12345583; doi:10.3390/ani15152257)
Supplement: Supplementary file 1 [file animals-15-02257-s001.zip › Supplementary Table S2.pdf]

Supplementary Table S2. Structural characterization of the identified compounds.

NO	Name	Formula	m/z	RT [min]	mzCloud best match	Area
1	L-Isoleucine	$C_6H_{13}NO_2$	132.1	2.46	99.9	107650991960
2	Uracil	$C_4H_4N_2O_2$	113.03	3.66	99.9	2519401763
3	L-Tyrosine	$C_9H_{11}NO_3$	182.08	3.05	99.8	15275164569
4	Dibutyl phthalate	$C_{16}H_{22}O_4$	279.16	18.53	99.8	344386141.3
5	Di(2-ethylhexyl) phthalate	$C_{24}H_{38}O_4$	391.28	23.02	99.6	506309940.2
6	(12Z)-9,10,11-trihydroxyoctadec-12-enoic acid	$C_{18}H_{34}O_5$	353.23	16.55	99.4	999439811.7
7	Azelaic acid	$C_9H_{16}O_4$	187.1	13.43	99.2	210628866.7
8	Stearic acid	$C_{18}H_{36}O_2$	283.26	23.95	99.2	120904655.6
9	Dipropyleneglycol dibenzoate	$C_{20}H_{22}O_5$	343.15	17.87	99.1	43223264.35
10	Citroflex 4	$C_{18}H_{32}O_7$	361.22	18.69	99.1	39731618.62
11	Linoleic acid	$C_{18}H_{32}O_2$	313.27	22.11	99.1	6328399376
12	Oleic acid	$C_{18}H_{34}O_2$	281.25	22.88	99.1	109557158.5
13	Benzoic acid	$C_7H_6O_2$	121.03	9.77	99	203745529.5
14	(2S,3S,4S,5R,6S)-3,4,5-trihydroxy-6-[(5-hydroxy-8-methoxy-4-oxo-2-phenyl-4H-chromen-7-yl) oxy] oxane-2-carboxylic acid	$C_{22}H_{20}O_{11}$	461.11	15.29	99	143043398.9
15	3-(propan-2-yl)-octahydropyrrolo[1,2-a]pyrazine-1,4-dione	$C_{10}H_{16}N_2O_2$	197.13	9.31	98.8	102077528.9
16	Dimethyl sebacate	$C_{12}H_{22}O_4$	231.16	14.68	98.8	210548234.5
17	4-Dodecylbenzenesulfonic acid	$C_{18}H_{30}O_3S$	325.18	19.77	98.8	178963035.9
18	Bis(3,5,5-trimethylhexyl) phthalate	$C_{26}H_{42}O_4$	419.31	24.16	98.7	243082887.4
19	3-(2-methylpropyl)-octahydropyrrolo[1,2-a]pyrazine-1,4-dione	$C_{11}H_{18}N_2O_2$	211.14	11.22	98.3	245075844.3
20	Ethylmalonic acid	$C_5H_8O_4$	131.03	6.17	97.5	1054003943
21	Bis(2-ethylhexyl)adipate	$C_{22}H_{42}O_4$	371.31	23.08	97.2	20397334.96
22	octadec-9-ynoic acid	$C_{18}H_{32}O_2$	263.24	21.42	97	1655020577
23	4-Aminophenol	C_6H_7NO	110.06	1.71	96.8	191641188.1
24	DL-Glutamine	$C_5H_{10}N_2O_3$	147.08	1.58	96.6	671758860.2
25	Diisodecyl phthalate	$C_{28}H_{46}O_4$	469.33	22.09	96.6	229299548.3
26	3-{2-[(1R,4aS,5R,6R,8aS)-6-hydroxy-5-(hydroxymethyl)-5,8a-dimethyl-2-methylidene-decahydronaphthalen-1-yl]-1-hydroxyethyl}-2,5-dihydrofuran-2-one	$C_{20}H_{30}O_5$	333.21	13.34	96	16408097.25
27	Octyl decyl phthalate	$C_{26}H_{42}O_4$	441.3	23.95	95.9	74812793.91
28	3-[(E)-2-[(1R,4aS,5R,6R,8aR)-6-hydroxy-5-(hydroxymethyl)-5,8a-dimethyl-2-methylidene-decahydronaphthalen-1-yl]ethenyl]-2,5-dihydrofuran-2-one	$C_{20}H_{28}O_4$	315.19	14.06	95.7	175170876.4
29	N-(5-acetamidopentyl)acetamide	$C_9H_{18}N_2O_2$	209.13	6.35	95	249276447
30	3-hydroxy-3-methylpentanedioic acid	$C_6H_{10}O_5$	185.04	1.14	94.5	6381867.082
31	Tridemorph	$C_{19}H_{39}NO$	298.31	23.78	93.6	12359249.76

32	1-{2,8-dioxo-1,7-diazatricyclo-dodecan-3-yl}propan-2-yl carbamate	$C_{14}H_{21}N_3O_4$	334.12	10.12	92.7	49440042.35
33	2,3-dihydroxypropyl 12-methyltridecanoate	$C_{17}H_{34}O_4$	285.24	20.65	92.6	18027167.1
34	L-Valine	$C_5H_{11}NO_2$	118.09	1.57	92	12171124120
35	Arachidonic acid	$C_{20}H_{32}O_2$	305.25	22.28	90.5	69342264.33
36	Genistein	$C_{15}H_{10}O_5$	269.05	20.25	90.2	57702049.11
37	1-(3-Trifluoromethylphenyl)piperazine	$C_{11}H_{13}F_3N_2$	231.11	8.44	89	82612106
38	Benzoic acid	$C_7H_6O_2$	121.03	12.26	86.4	92440541.59
