# Supplementary material for: Evaluation of Immunoprotective Activities of White Button Mushroom (Agaricus bisporus) Water Extract Against Major Pathogenic Bacteria (Aeromonas hydrophila or Vibrio fluvialis) in Goldfish (Carassius auratus)
Source: Animals (Basel). 2025 Aug 1;15(15):2257. doi: 10.3390/ani15152257 (PMC12345583; doi:10.3390/ani15152257)
Supplement: Supplementary file 1 [file animals-15-02257-s001.zip › Supplementary Table S3.pdf]

Supplementary Table S3. The LD_{50} determination of *A. hydrophila* and *V. fluvialis* in *C. auratus*.

Bacteria	Bacterial dose (CFU)	No.	Death, no.	Survival, no.	ADR, %
A. hydrophila	4×10^8	10	3	7	30
	8×10^8	10	5	5	50
	10×10^8	10	8	2	80
	12×10^8	10	10	0	100
V. fluvialis	6×10^8	10	2	8	20
	8×10^8	10	4	6	40
	1×10^9	10	5	5	50
	2×10^9	10	9	1	90

Note: ADR, accumulating death rate. the LD_{50} of *A. hydrophila* or *V. fluvialis* were 8×10^8 CFU and 1×10^9 CFU, respectively.
